# Can sensory and semantic priming enhance the effects of guided self-compassion meditation? A proof-of-concept study

**DOI:** 10.3389/fpsyg.2024.1385799

**Published:** 2024-08-27

**Authors:** Ivana Buric, Maja Wrzesien, Jelica Milojičić, Anna Ridderinkhof, Esther De Bruin, Susan Bögels

**Affiliations:** ^1^Department of Psychology, University of Amsterdam, Amsterdam, Netherlands; ^2^Instituto Polibienestar, University of Valencia, Valencia, Spain; ^3^Department of Developmental Psychopathology, University of Amsterdam, Amsterdam, Netherlands; ^4^Department of Psychology, University of Belgrade, Belgrade, Serbia; ^5^Research Institute of Child Development and Education, University of Amsterdam, Amsterdam, Netherlands; ^6^Center UvA Minds, University of Amsterdam, Amsterdam, Netherlands

**Keywords:** self-compassion, sensory priming, semantic priming, meditation, self-criticism

## Abstract

**Introduction:**

Self-compassion is a fundamental aspect of psychological health and well-being that can be cultivated through self-compassion meditations, but it remains unclear how to facilitate this most effectively. This study is the first to explore whether sensory and semantic priming introduced prior to a guided self-compassion meditation could enhance the effects of meditation in comparison with a control condition.

**Methods:**

The study was conducted with 3 × 3 repeated measures between-group design, including three groups (sensory priming, semantic priming and control group), and three assessment time points of state self-compassion, self-criticism, and positive and negative affect (at baseline, after priming, and after guided meditation). Additionally, a meditation appeal questionnaire was used. The total sample size included 71 students who underwent a 3-min priming intervention followed by a 15-min self-compassion guided meditation session.

**Results:**

First, prior to guided meditation, sensory priming significantly decreased state self-criticism more than the control condition or semantic priming, although some reliability issues of the applied self-criticism scale must be taken into consideration. Second, neither sensory nor semantic priming changed state self-compassion, positive affect or negative affect. Third, neither semantic nor sensory priming significantly enhanced the effects of guided self-compassion meditation either in positive and negative affect, self-compassion states, self-criticism states, or in the appeal of the meditation experience.

**Discussion:**

Although this study is underpowered (estimated post hoc power ranges from 0.20 to 0.42), the findings provide preliminary insights into the potential priming has as a tool to enhance meditation effects and provide guidelines for future studies.

## 1 Introduction

Clinical and research interest in self-compassion has gained popularity in the past decade due to its vast array of positive outcomes that are relevant to public health, including reduced psychopathology (MacBeth and Gumley, 2012; Kirby et al., 2017), increased well-being (Zessin et al., 2015), and coping with stress (Ewert et al., 2021). Self-compassion can be defined as a “cognitive, affective, and behavioral process” directed toward the self that includes five elements: recognizing suffering, understanding its universality, feeling empathy and emotional resonance, tolerating uncomfortable feelings and motivation to act to alleviate suffering (Strauss et al., 2016). The capacity for self-compassion varies among individuals and it is not fixed; instead, it remains flexible throughout the lifespan (Neff, 2003). As such, self-compassion training is a crucial component of several evidence-based therapeutic modalities, such as a Compassion Focused Therapy (Gilbert, 2009) or Mindful Self Compassion (Neff and Germer, 2013), which are effective for mental health across non-clinical, clinical, and subclinical populations (Kirby et al., 2017; Wilson et al., 2019). This transdiagnostic effectiveness is not surprising, given that self-compassion targets maladaptive mechanisms contributing to psychopathology such as self-criticism (Clark et al., 1994; Gilbert and Procter, 2006; Gilbert, 2009; Wakelin et al., 2022) and enhances emotion regulation (Porges, 2017; Inwood and Ferrari, 2018). As one of the pioneering clinicians in the field of compassion mentioned, “The field of self-compassion in therapy is currently in its adolescence” (Germer, 2023). In other words, until now the majority of studies have focused on validating compassion-based interventions in a specific context (e.g., population or disorder), while little is known about how to optimize the effectiveness of this practice.

Self-compassion practice typically includes guided meditation, which directs attention inward while using mental imagery to induce feelings of warmth, nurture and benevolence towards the self (Gilbert, 2020). Indeed, self-compassion meditations can be categorized as the constructive type of meditative practices (Dahl et al., 2015) where the quality of the meditation practice can be influenced by the practitioner’s ability to generate mental imagery (Navarrete et al., 2021; Wilson-Mendenhall et al., 2023), and by the activation of the somatosensory component to elicit and sustain compassion (Navarrete et al., 2021). Although mental imagery plays an important role in the self-compassion meditation practice, finding different ways to enhance the somatosensory component by evoking an inner sense of safety that arises from the soothing system is another crucial factor that influences the quality of this meditation practice (Gilbert, 2009; Navarrete et al., 2021). The soothing system, one of the three emotion regulation systems as conceptualized by Gilbert in Compassion-Focused Therapy, is characterized by the activation of the parasympathetic nervous system, fostering feelings of safety, warmth, and comfort (Gilbert, 2009, 2020). By cultivating this system, individuals can effectively regulate their emotions and navigate challenges (Gilbert, 2009, 2020; Porges, 2017; Inwood and Ferrari, 2018). Discovering new ways to activate the soothing system, especially its somatosensory component, might further improve the effectiveness of self-compassion practice and of compassion-based therapeutic modalities. However, to the authors’ knowledge, the only existing attempts to trigger the soothing system in the context of self-compassion meditation have been done pharmacologically. For instance, Kamboj et al. showed that 3,4-Methylenedioxymethamphetamine (MDMA, commonly known as ecstasy) can enhance the effects of self-compassion meditation by further increasing self-compassion and decreasing self-criticism (Kamboj et al., 2015, 2018). On the other hand, Rockliff and her colleagues (Rockliff et al., 2011) showed that intranasal oxytocin can increase the ease of compassionate mental imagery, but this effect was smaller in people with low attachment security and high self-criticism. Since the use of pharmacological interventions in combination with meditation as a regular enhancement tool for meditation could be problematic due to its possible adverse effects (especially in the absence of medical guidance), its temporary action, and still not well-understood long-term consequences (Buchert et al., 2003), other ways to activate the soothing system should be considered a priority.

Drawing from social psychology, one non-pharmacological approach to activating the soothing system could be performed through priming. Indeed, priming consists in modifying the quality, intensity or duration of emotional responses in an implicit way (i.e., without explicit intentions). This implicit activation (i.e., priming) refers to the activation of mental representations through exposure to stimuli, which then influences subsequent experiences (Shalev and Bargh, 2011; Molden, 2014). For instance, semantic priming via exposure to words such as “wrinkles” or “Bingo” (i.e., stimulus) can activate the elderly stereotype (i.e., mental representations), leading to behavior changes such as walking more slowly upon exiting the laboratory (Bargh et al., 1996). Despite failed replication attempts of many priming studies (Cesario, 2014; Molden, 2014), some are supported by a substantial body of research, including numerous meta-analyses (Zessin et al., 2015; Kirby et al., 2017; Ewert et al., 2021), affirming the robustness of priming effects. However, the effects of priming on self-compassion meditation have not yet been studied. Applying priming within self-compassion interventions could increase readiness to practice self-compassion techniques, boost their effectiveness, or increase adherence to intervention instructions (Shalev and Bargh, 2011). As such, priming could be a valuable tool to enhance the effects of guided self- compassion meditation by activating the soothing system and creating a sense of safety, warmth, and comfort. For individuals who struggle to activate the soothing system, such as those with interpersonal trauma or an avoidant attachment style, finding the means to activate the soothing system is necessary so that they can gain benefits from self-compassion meditation that they might otherwise perceive as difficult, ineffective, or even threatening.

To our knowledge, only one study directly tested the effects of priming on outcomes of meditation (Rowe et al., 2016). While they did not specifically evaluate self-compassion meditation nor how priming influences the immediate effect of meditation, they tested and confirmed that semantic priming prior to mindfulness meditation can increase the willingness to commit to regular mindfulness practice (Rowe et al., 2016). Our study is the first to examine the potential benefits of priming and its enhancing effect on immediate outcomes of self-compassion meditation. By priming individuals with stimuli related to the soothing system through sensory or semantic cues, it is hypothesized that guided self-compassion meditation will be more effective than it is when priming is not present. Here we use two priming modalities, one that can be defined as a “bottom-up” priming (activating the soothing system in response to a sensory stimulus), and one that can be defined as “top-down” (activating the soothing system in response to an explicit cognitive stimulus) (Strauss et al., 2016). The first priming modality is sensory priming, which includes holding a warm therapeutic pad in a fluffy cover and this type of warmth-based priming has been used previously in other studies related to pro-sociality (Neff, 2003) and trust (Gilbert, 2009), but not in mediation research. The second priming modality is semantic priming, which includes an unscrambled sentence task and has also only been used in different research areas related to emotion regulation until now (Neff and Germer, 2013). The specific objectives of this study are twofold: (1) to test whether sensory and semantic priming on its own is effective (i.e., can increase self-compassion state and positive affect, and decrease self-criticism state and negative affect), (2) to test whether sensory and semantic priming introduced prior to a guided self- compassion meditation can enhance the effects of meditation and lead to greater increases in self-compassion state and positive affect, decreases in self-criticism state and negative affect, as well as greater appeal to meditation in comparison with a control group that does not receive any priming prior to meditation instructions. The choice of the outcome measures is based on Social Mentality Theory that differentiates between self-compassion as a state that involves caregiving and care-seeking, and between self-criticism as a state that has a function to protect us from social threats (Wilson et al., 2019). Both self-compassion and self-criticism can be considered as “complex cognitive, emotional, motivational, and behavioral responses to the self” that have a particular temporal relationship which has been rarely studied together (Wakelin et al., 2022). The choice of positive and negative affect as outcome measures stems from a meta-analysis that found significant small indirect effects of self-compassion on health behaviors through both positive and negative affect (Sirois et al., 2015), which is also in line with Neff’s model (Neff, 2003) that posits that self-compassion is linked to positive affect and it underscores the importance of examining affective states as outcomes in self-compassion interventions. Investigating the combined effects of priming and self-compassion meditation in this study contributes to a deeper practical understanding of how to facilitate self-compassion effectively. The findings of this study not only have important implications for clinical practice, but also set the foundations for future research on priming and meditation.

## 2 Materials and methods

### 2.1 Participants

A total of 71 students (75.4% female; mean age of 26.64 years, SD = 5.67) recruited from the University of Amsterdam participated in the study and were awarded 5€ for study participation. Additionally, a lottery of 50 € coupon among all participants was performed. Inclusion criteria included English-speaking adults between 18 and 45 years old with no history of severe psychiatric disorders nor previous experience in self-compassion meditation. Informed consent was obtained from all participants prior to their participation in the study.

### 2.2 Procedure

**FIGURE 1:**
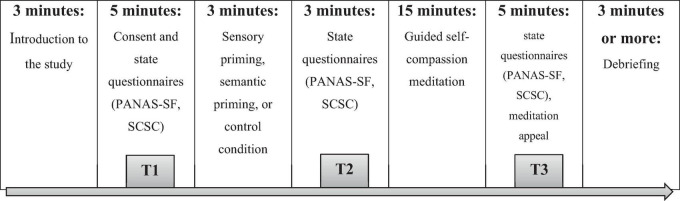
A graphical representation of the lab visit including three timepoints of measurements (T1, T2, T3).

The study employed 3x3 repeated measures between-group design, with three assessment time points (baseline, after priming, and after guided self-compassion meditation) and three groups (sensory priming, semantic priming and control group). Testing was done during one individual lab session (in closed independent cubicles without the presence of the researcher) that lasted approximately 35 min per participant (see Figure 1). Following the arrival at the lab, participants were informed about the study procedures and aims (i.e., studying how different priming modalities affect the meditation experience). The participants signed the consent form and completed questionnaires that examined their baseline state (self-compassion and self-criticism states, and positive and negative affect). Participants were then randomly allocated to one of the three conditions: semantic priming, sensory priming, or a control condition. The sensory priming group received a 3-min sensory priming intervention, which involved holding a warm therapeutic pad in a fluffy cover based on previous research (Kang et al., 2011; Storey and Workman, 2013). The semantic priming group received a 3-min priming intervention, which involved an unscrambled sentence task based on previously tested methodology (Williams et al., 2009) where participants had to construct 10 sentences from four scrambled words (e.g., “*The sand is warm”, “Her touch is tender*”), in which 6 sentences included affiliative system activating word such as (warm, tender, loving). The control group did not receive any priming, but instead, participants did a set of simple hand mobility and strength exercises for the same duration as the priming conditions to control for factors such as engagement in a task, duration of time spent alone in a cubicle and physical activity comparable to writing or holding a pad. Following the priming intervention, participants again completed the measures of self-compassion state, self-criticism state, and positive and negative affect. They then listened to a recording of a 15-min guided self-compassion imagery meditation suitable for beginners (i.e., Compassionate Friend from the MSC program) (Neff and Germer, 2013). After the guided meditation, participants completed the final set of questionnaires. Additionally, a meditation appeal questionnaire was administered to assess participants’ subjective experience of the meditation session. At the end of the lab session, participants were thanked and debriefed. Finally, participants were examined for any suspicions regarding the experimental objectives, a recommended procedure in priming (Bargh and Chartrand, 2000). None of the participants expressed any suspicion regarding the intentions behind the priming and task procedures.

## 3 Outcome measures

### 3.1 Demographics

A brief questionnaire included standard demographic questions including age, gender and ethnicity.

### 3.2 Positive and negative affect

The Positive and Negative Affect Schedule Short Form (PANAS-SF) consists of two 10-item scales developed to assess positive and negative affect (Thompson, 2007). This version contains five items for positive affect (“active/determined/attentive/inspired/alert”) and five items for negative affect (“afraid/nervous/upset/hostile/ashamed”). For each item participants are instructed to indicate on a scale of 1 (“Very slightly or not at all”) to 5 (“Extremely”) how well the item described their current state. The results provide separate scores for positive affect and negative affect. Both positive and negative scales of the PANAS-SF have shown adequate reliability (Cronbach’s alpha of 78 and 0.76, respectively) and it is a valid measure of affect across countries (Thompson, 2007). In this study the scales were equally reliable (positive affect: T1: α = 0.89, T2: α = 0.92; T3: α = 0.90. and negative affect T1: α = 0.76, T2: α = 0.90, T3: α = 0.81). The measure was selected as a traditionally used self-report emotional experience outcome, previously used in the priming studies (Yuan et al., 2015).

### 3.3 State self-compassion and self-criticism

The Self-Compassion and Criticism Scale (SCCS) is a scenario-based trait measure and its adapted state measure (Falconer et al., 2015). The state measure consists of three items (self-reassurance, self-soothing, self-compassion) that correspond to self-compassion subscale, and three items (self-contempt, self-criticism and self-harshness) that correspond to the self-criticism subscale, that are rated on a 1 (“Not at all”) to 7 (“Highly”) Likert scale. The SCCS has shown good reliability of.87 and.91 for self-criticism and self-compassion subscales, respectively (Falconer et al., 2015). In our study, while the self-compassion subscale has shown adequate reliability in all three time points (respectively: α = 0.67, α = 0.98, α = 0.84), the self-criticism obtained low reliability in the first time point, but not in the second and third (respectively: α = 0.41, α = 0.75, α = 0.78).

### 3.4 Meditation appeal questionnaire

A meditation appeal questionnaire was adapted from two previous studies (Rockliff et al., 2011; Rowe et al., 2016). It contains ten items (answered on a scale from 1 to 10) that assess participants’ subjective experience after the meditation, including their perceived easiness, resistance, difficulties, emotionality, mind wandering, and intention to practice again. Psychometric properties are not available, thus we used this questionnaire for exploratory purposes only.

## 4 Data analyses

Non-parametric analyses were conducted using IBM SPSS Statistics (Version 22 for Windows) because the assumption for parametric group comparisons were not met (i.e., deviations from normal distribution and violation of homogeneity of variance assumption in several variables). First, we tested for outliers using the Mahalanobis distance that showed two participants were outliers, and these participants were excluded from all the analyses. Second, baseline differences between the three randomized groups were checked with Kruskal-Wallis to test if randomization was successful before continuing with further analysis. Next, to investigate the combined effect of priming and self-compassion meditation (T3) on state self-compassion, self-criticism, and positive and negative emotions across different priming conditions (sensory, semantic, and control), we conducted multiple Kruskal-Wallis tests. We calculated change scores by deducting the final score on each scale from the first, baseline score (T3–T1) and compared all groups. To investigate solely the effect of priming on state self-compassion, self-criticism, and positive and negative emotions across different priming conditions, we again conducted multiple Kruskal-Wallis tests and in this case, we calculated the change scores between priming and baseline conditions (T2–T1). Whenever the Kruskal-Wallis tests found significant differences between the three groups (i.e., *p*-values adjusted for multiple comparisons were smaller than 0.05), pairwise comparisons were done to detect between which two groups the detected difference occurred.

## 5 Results

Below we show the results of our analyses that should only be considered as preliminary and hypotheses generating due to limited sample size and thus insufficient statistical power (post hoc power was calculated in GPower using effect sizes based on the two most relevant meta-analyses (Lucas, 2000; Gillath et al., 2022), and showed the power in our study ranges from 0.20 to 0.42). There were no differences between the three groups at baseline (T1) in self-compassion (*H* = 1.700, df = 2, *p* = 0.428) self-criticism (*H* = 0.027, df = 2, *p* = 0.987), positive affect (*H* = 0.849, df = 2, *p* = 0.654) and negative affect (*H* = 0.253, df = 2, *p* = 0.881) (see Supplementary Table 1 in the Supplementary materials), hence we could continue with further analysis. The results of the analysis that tested the first study aim—whether sensory and semantic priming on their own is effective—suggested that there was an increase in state self-compassion with a medium effect size, a decrease in state self-criticism with a large effect size, and no significant changes in positive or negative affect (see Table 1). To explain the observed significant group differences in self-compassion and self-criticism, pairwise comparisons were run and suggested that a significant difference in self-compassion occurred between sensory and semantic priming groups (*H* = 16.512, SE = 5.973, η^2^ = 0.220, *p* < 0.05^1^), where sensory priming increased self-compassion more than semantic priming (Mean Rank Sensory = 43.58, Mean Rank Semantic = 27.07, Mean Rank Control = 33.35). However, sensory priming did not increase self-compassion more than the control group. On the other hand, when it comes to self-criticism, pairwise comparisons suggested a significant difference between sensory priming and the control group (*H* = −15.104, SE = 5.733, η^2^ = 0.199, *p* < 0.05)—sensory priming decreased self-criticism more than the control group and the effect size is large. There was also a significant difference between sensory priming and semantic priming (*H* = −19.292, SE = 5.935, η^2^ = 0.262, *p* < .05*^i^*), but semantic priming was not significantly more effective than the control group (Mean Rank Sensory = 23.88, Mean Rank Semantic = 43.17, Mean Rank Control = 38.98; see Supplementary Figure 1 in the Supplementary materials). Together, these results suggest that neither sensory nor semantic priming is more effective than the control group in terms of changing state self-compassion, but that sensory priming can effectively decrease state self-criticism.

**TABLE 1:** The effects of priming on main outcome measures.

Change score (T2−T1)	H	*p* ^a^	Median	IQR	*η*2 b
Self-compassion	7.892	0.019*	0.333	−0.333–1.167	0.089
Self-criticism	12.044	0.002*	0.333	0.000–0.750	0.152
Positive affect	0.140	0.933	0.000	−0.200–0.200	0.028
Negative affect	5.686	0.058	0.000	−0.200–0.000	0.056

^a^Adjusted *p*-values (multiple comparisons, Bonferroni adjustment),

* < 0.05 is considered significant.

^b^Partial eta square represents effect sizes [η^2^ = (H–k + 1)/(n−k)].

The results of the analysis that tested the second study aim, which is the combined effects of priming and self-compassion meditation across all three groups, suggested no significant results on either state self-compassion, self-criticism, positive affect, or negative affect (Table 2). When testing the differences between T3 and T1 in the control group alone, there were no significant results in any of the tested variables self-compassion (*z* = −0.437; *p* = 0.662), self-criticism (*z* = −1.543, *p* = 0.123), positive affect (*z* = −0.163; *p* = 0.871), and negative affect (*z* = −0.425, *p* = 0.671). These results suggest that sensory or semantic priming introduced prior to a guided self-compassion meditation does not enhance the effects of meditation, and also show that independent effects of self-compassion were not significant because there were no changes in the control condition that was exposed to hand exercises instead of priming. Finally, we expected that the appeal to the meditation would be significantly greater in both semantic and sensory priming groups compared to the control condition. For this, we used an ad hoc and non-validated questionnaire, so we provided analyses on each item from this questionnaire. As can be seen in Table 3, none of the results are significant therefore there are no differences between groups.

**TABLE 2:** The combined effect of priming and self-compassion meditation on main outcome measures.

Change score (T3– T1)	*H*	*_*p*_* ^a^	Median	IQR	*_η_*2 b
Self-compassion	4.726	0.094	0.000	−1.000–1.000	0.041
Self-criticism	4.747	0.093	0.000	−0.083–0.833	0.042
Positive affect	1.528	0.466	0.000	−0.225–0.500	0.007
Negative affect	0.929	0.628	0.000	−0.400–0.000	0.016

^a^Adjusted *p*-values (multiple comparisons, Bonferroni adjustment), < 0.05 is considered significant.

^b^Partial eta square represents effect sizes [η^2^ = (*H*–*k* + 1)/(*n*– *k*)].

**TABLE 3:** Group differences in exploratory per-item analysis of the mediation appeal questionnaire.

	*H*	*p*	Median	IQR	*_η_*2
Easiness receiving compassion	1.179	0.555	6.000	4.000–7.500	0.012
Wanting to resist	0.747	0.688	5.000	3.000–8.000	0.019
Tension during meditation	0.615	0.735	3.000	2.000–6.000	0.021
Trying to create a visual image	2.382	0.304	7.000	6.000–8.000	0.006
Clearness of image	2.151	0.341	7.000	6.000–8.000	0.002
Moved by image	1.975	0.372	7.000	5.000–8.000	0.000
Intention to practice	1.148	0.563	7.000	5.000–8.000	0.013
Sadness feelings during meditation	1.775	0.412	4.000	1.000–7.000	0.003
Mind wandering	0.996	0.608	6.000	4.000–7.000	0.015
Evaluation of experience	1.688	0.430	2.000	1.000–3.000	0.005

## 6 Discussion

This proof-of-concept study aimed to investigate whether sensory and semantic priming could enhance the effects of guided self-compassion meditation and yielded several findings. However, due to low statistical power, all findings must be considered preliminary until they are replicated in larger studies because there is a higher probability that non-significant findings could be due to an insufficient sample size rather than the absence of a true effect. First, sensory priming had a significant and large effect on reducing state self-criticism compared to semantic priming and the control group. Second, sensory priming did not significantly increase self-compassion or positive affect compared to the other conditions, nor decrease negative affect. Finally, neither sensory nor semantic priming significantly enhanced the effects of guided self-compassion meditation in terms of positive and negative affect, self-compassion states, self-criticism states, or the appeal of the meditation experience.

The finding that sensory priming reduced self-criticism suggests that sensory priming may be a more promising tool for reducing self-critical thoughts instead of directly targeting self-compassion. This goes in line with previous studies emphasizing the importance of experiencing internal scripts based on warmth, compassion and forgiveness when targeting self-criticism (Gilbert et al., 2006; Gilbert, 2009). For instance, holding a warm and fluffy pad during therapeutic work might implicitly facilitate these warmth-based internal scripts that have an impact on self-criticism, which remains to be tested in future studies. As mentioned above, the finding that sensory priming decreases state self-criticism is not robust because the reliability of the self-criticism subscale was low in our sample at the first time point (T1, baseline assessment). Previous studies using the SCCS scale reported only the reliability from the original paper (Falconer et al., 2015), without reporting reliability based on their data (Kamboj et al., 2015; Falconer et al., 2016; Serpell et al., 2020; Hidding et al., 2024). Therefore, because the low reliability of the self-criticism subscale was observed in our study in one out of the three timepoints, the results might not accurately represent the true relationship between sensory priming and self-criticism, instead the observed effect could be influenced by measurement error. For this reason, future studies should directly test this relationship on a larger sample and with a more reliable instrument, pre-registration, and open data sharing, until then precise clinical recommendations cannot be made.

The finding that neither sensory nor semantic priming enhanced the effects of guided self-compassion meditation is inconsistent with previous research suggesting that priming can enhance the effects of psychological interventions (Shalev and Bargh, 2011) and that priming can enhance willingness to practice mindfulness meditation (Rowe et al., 2016). This lack of significant effects on positive and negative affect, self-compassion states, self-criticism states, and the appeal of the meditation experience could be due to various factors. First, the duration of priming was 3 min long, which may not have been sufficient to produce significant changes in the soothing system and induce a feeling of inner safety and inner warmth. Longer priming sessions or other types of priming might be more effective in boosting the effects of self-compassion. Second, it might also be that neither semantic nor warmth-based priming influences the internal scripts related to the soothing system. In this line, although not directly related to the meditation outcomes, a recent meta-analytical review shows little support for the temperature (i.e., warmth-priming) effect on pro-sociality (Serpell et al., 2020). Additionally, we must also note that the brief self-compassion meditation that was employed in this study was not effective, which is seen through non-significant changes in outcome variables in the control group that had hand exercises instead of priming. This ineffectiveness of the self-compassion meditation itself could have prevented any priming effects from emerging, thus limiting the generalizability of our results. Even though the implemented short meditation was extracted from a validated protocol (Mindful Self Compassion) it is only a 15-min meditation that might not induce expected outcomes in novice meditators (i.e., a decrease in self-criticism and negative affect, and increase in positive affect and self-compassion). According to recent meta-analytic results (Schumer et al., 2018) short-term meditations present some potential for decreasing negative affect, however the authors also suggest that given the presence of publication bias in this research field more published studies are needed. Thus, the non-significant results in this manuscript also contribute to decreasing the publication bias in this research area, and applying different meditations in future priming studies might lead to different results. Furthermore, as mentioned earlier, this study did not manage to recruit a sufficient number of participants to achieve sufficient statistical power to detect smaller effects, which could be one of the reasons of non-significant results that were observed. Another important limitation of this study is the exclusive use of self-report measures in this study. While self-report measures are commonly used in self-compassion and in priming research, they are subject to biases and may not fully capture the nuanced changes in the soothing system when they occur. Including physiological measures such as heart rate variability; and/or implicit self-report measures such as the implicit version of the positive and negative affect scale (Quirin et al., 2009) could provide additional data on the effects of priming and guided self-compassion meditation in future studies. Especially since other priming studies demonstrated that the effect of semantic priming of emotional regulation was effective only on the implicit, physiological level (Yuan et al., 2015). However, even with optimal research design and methodology, there is a possibility that there will be inconsistencies across future studies that examine either the effects of priming on state outcomes or the combined effect of priming and self-compassion meditation on state outcomes. Overall, the robustness and generalizability of priming effects have been questioned because priming studies are often not replicated or replication studies find different effects (Cesario, 2014; Molden, 2014). The field has also grappled with the lack of standardized methodologies, leading to variability in experimental designs and difficulty in comparing findings across studies (Cesario, 2014; Molden, 2014).

Nevertheless, priming holds promise as a means to activate the soothing system, and potentially also in enhancing the effects of guided self-compassion meditation and other types of meditation. Further research is needed to understand how to use priming and under what conditions it can boost the effects of meditation. First, we can expect there will be differential effects of priming on beginner and experienced meditators. Beginners may require more explicit and prolonged priming interventions to establish a sense of safety, as they may lack the internal resources and familiarity with meditation practices to readily access this state. On the other hand, experienced meditators may benefit from more subtle and brief priming interventions, as they have already developed a certain level of proficiency in cultivating a sense of safety during meditation. Moreover, individual differences such as type of attachment style and level of social safety, that could not be tested due to the sample size in this study, could also influence the practice. Understanding these differential effects can inform the development of tailored priming interventions based on the personal characteristics of the participant. Second, the optimal type and dosage of priming necessary to boost the effects of meditation remains unknown. This study tackled sensory and semantic priming that targets the soothing system and is 3 min long, while other types of priming or longer duration might be more effective in boosting the effects of self-compassion meditation. Should priming be a one-time event before meditation, or would multiple sessions yield more sustained effects? Should the duration of priming be prolonged to allow for a deeper sense of safety to be established, or would shorter bursts be equally effective? Answering these questions will provide valuable insights into the practical implementation of priming techniques in meditation practices. Finally, exploring different ways in which individuals activate their soothing system outside of the laboratory to inform future interventions could also be an interesting path. For instance, methodologies based on ecological momentary assessment [e.g., Gilbert et al., 2006] or social media post analysis (Ziemer, 2022) could bring more insights into possible ecological mechanisms of action. Overall, this proof-of-concept study provides valuable insights into the potential of priming modalities to enhance the effects of guided self-compassion meditation. However, it did not find significant effects of priming on self-compassion or the enhancement of guided self-compassion meditation, which could be due to a lack of statistical power to detect these effects. Future research should build upon these findings by conducting larger and more refined studies to further explore the effects of priming modalities on self- compassion and identify the most effective strategies for combining priming and meditation.

## Data availability statement

The datasets presented in this study can be found in online repositories. The names of the repository/repositories and accession number(s) can be found below: https://osf.io/gznp9.

## Ethics statement

The study received ethical approval from the Ethics Review Board of the Faculty of Social and Behavioral Sciences of the University of Amsterdam (2016-CDE-7472). The studies were conducted in accordance with the local legislation and institutional requirements. The participants provided their written informed consent to participate in this study.

## Author contributions

IB: Writing−review and editing, Writing−original draft, Visualization, Formal analysis. MW: Writing−review and editing, Resources, Project administration, Methodology, Investigation, Funding acquisition, Conceptualization. JM: Writing−review and editing, Software, Formal analysis, Data curation. AR: Writing−review and editing, Methodology, Investigation, Conceptualization. ED: Writing−review and editing, Supervision, Methodology, Investigation, Conceptualization. SB: Writing−review and editing, Supervision, Methodology, Investigation, Conceptualization.
