# Supplementary material for: Can sensory and semantic priming enhance the effects of guided self-compassion meditation? A proof-of-concept study
Source: Front Psychol. 2024 Aug 27;15:1385799. doi: 10.3389/fpsyg.2024.1385799 (PMC11385674; doi:10.3389/fpsyg.2024.1385799)
Supplement: Supplementary file 1 [file Table_1.DOCX]

**Table S1.** Demographic variables presented across all three groups with tests for baseline differences between groups

|  | **Sensory priming group** (N = 24) | **Semantic priming group**  (N = 21) | **Control group**  (N = 24) | **Test statistic** | **p** |
| --- | --- | --- | --- | --- | --- |
| **Age** |  |  |  | .30 | .86 |
| Mean ± SD | 25.42 ± 7.28 | 23.62 ± 3.93 | 24.75 ± 5.202 |  |  |
| Min-Max | 18.00-46.00 | 18.00-34.00 | 19.00-38.00 |  |  |
| **Gender (%)** |  |  |  | .36 | .83 |
| Female | 79.17 | 71.43 | 75.00 |  |  |
| Male | 20.83 | 28.57 | 25.00 |  |  |
| **Education (%)** |  |  |  | 9.45 | .49 |
| High school | 45.83 | 57.14 | 29.17 |  |  |
| Bachelor degree | 41.67 | 28.57 | 45.83 |  |  |
| Master degree | 9.52 | 9.52 | 16.67 |  |  |
| Doctoral degree | 0.00 | 0.00 | 4.17 |  |  |

**Figure S1.** Histogram with mean scores for: A) Self-compassion, B) Self-criticism, C) Positive Affect, D) Negative Affect presented across all three timepoints (T1-baseline, T2-after priming or control condition, T3-after meditation) and across all three groups

| 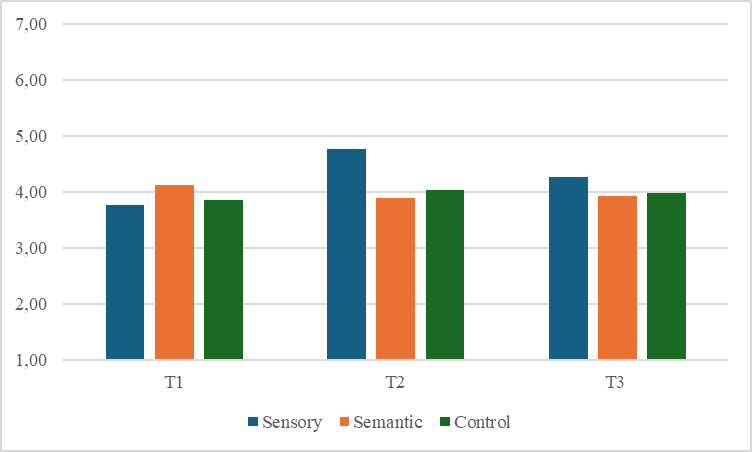  A | 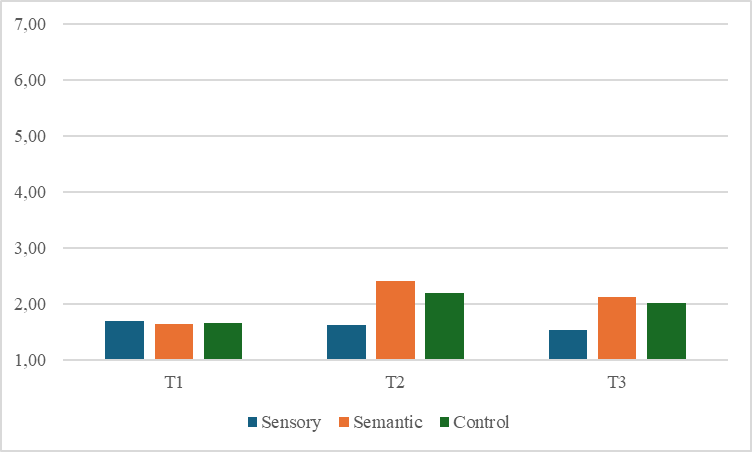  B |
| --- | --- |
| *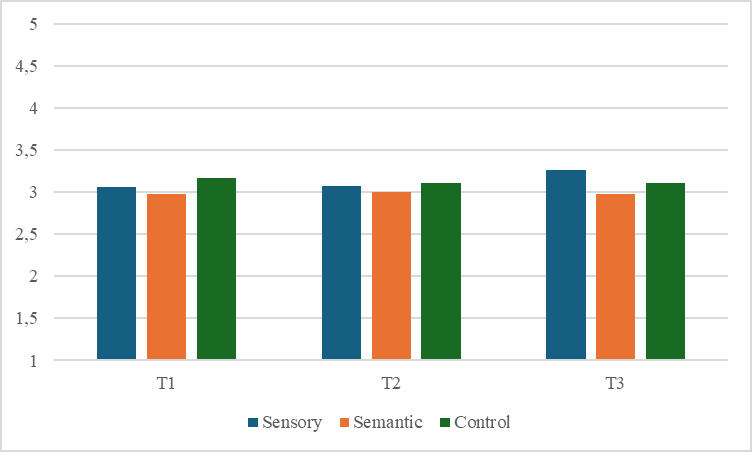*  C | 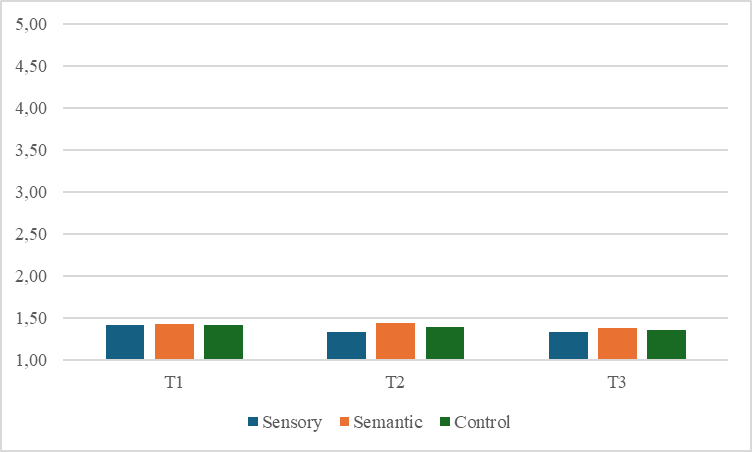  D |
